# Biophysical Measurement of the Balance of Influenza A Hemagglutinin and Neuraminidase Activities*

**DOI:** 10.1074/jbc.M114.622308

**Published:** 2015-01-13

**Authors:** Donald J. Benton, Stephen R. Martin, Stephen A. Wharton, John W. McCauley

**Affiliations:** From the Divisions of ‡ Virology and; § Physical Biochemistry, Medical Research Council National Institute for Medical Research, London NW7 1AA, United Kingdom

**Keywords:** Biophysics, Enzyme Turnover, Influenza Virus, Kinetics, Neuraminidase, Hemagglutinin, Receptor Analogs, Receptor Binding

## Abstract

The interaction of influenza A viruses with the cell surface is controlled by the surface glycoproteins hemagglutinin (HA) and neuraminidase (NA). These two glycoproteins have opposing activities: HA is responsible for binding the host receptor (sialic acid) to allow infection, and NA is responsible for cleaving the receptor to facilitate virus release. Several studies have demonstrated that compatible levels of HA and NA activity are required for a virus to replicate efficiently. This is consequently of great interest for determining virus transmissibility. The concurrent role of these two proteins in receptor binding has never been directly measured. We demonstrate a novel biophysical approach based on bio-layer interferometry to measure the balance of the activities of these two proteins in real time. This technique measures virus binding to and release from a surface coated with either the human-like receptor analog α2,6-linked sialic acid or the avian-like receptor analog α2,3-linked sialic acid in both the presence and absence of NA inhibitors. Bio-layer interferometry measurements were also carried out to determine the effect of altering HA receptor affinity and NA stalk length on receptor binding.

## Teaser

**Background:** Influenza A viruses contain the surface glycoproteins hemagglutinin (HA) and neuraminidase (NA), responsible for receptor binding and virus release, respectively.

**Results:** The contribution of HA and NA to virus interactions with receptor-coated surfaces was measured using bio-layer interferometry.

**Conclusion:** The balance between the activities of the two glycoproteins controls virus-cell interactions, therefore transmissibility.

**Significance:** This technique can be used to examine factors underlying emergent virus transmissibility.

## Introduction

Understanding the transmissibility of influenza A viruses (IAVs)^2^ is vital to monitoring new and emerging viral variants and understanding previous pandemics and their emergence. A major factor underlying IAV transmissibility is the interaction of the virus with the cell surface, which is mediated by two virus surface glycoproteins: hemagglutinin (HA) and neuraminidase (NA). HA acts as the receptor-binding protein, binding to the cell-surface receptor (sialic acid), and is a major factor in determining the host restriction of IAVs. The receptor specificity of HA is attuned to the sialic acid linkage present at the primary site of infection. Avian viruses typically bind to α2,3-linked sialic acid, and human viruses bind primarily to α2,6-linked sialic acid (1). NA is a sialidase and therefore acts as a receptor-destroying enzyme. NA is responsible for cleaving sialic acid, to which the virus may be bound, at various points in the infection cycle. This cleavage can result in release of the virus from the cell surface after infection, prevention of aggregation by virus binding to the glycoproteins of nearby viruses (2, 3), or release of the virus from sialic acid-rich mucins (4, 5).

It has long been hypothesized that there is a balance between the functions of HA and NA. Both the receptor-binding avidity of HA and the receptor-destroying activity must be in the correct proportions for the virus to efficiently infect and transmit (6). These HA- and NA-mediated receptor interactions are controlled by a number of physical characteristics of the virus and associated proteins: 1) HA receptor-binding affinity (*K_d_*); 2) NA kinetic characteristics (*K_m_* and *k*_cat_); 3) the relative proportions and distribution of HA and NA on the virus surface; 4) virus morphology, which could alter protein distribution; and 5) the accessibility of NA to the substrate, which can be modulated by altering the length of the stalk between the catalytic region of the protein and the viral membrane.

Extensive studies in the past have shown an interdependence of HA and NA activities. These studies are generally related to altering the activity of HA or NA and observing the relative infectivity or compensatory mutations that occur after passage. Studies involving the inhibition of NA with inhibitors have shown that escape mutants can be generated with substitutions in HA alone (7–9), and sensitivity to these inhibitors is determined by both HA and NA (3). These substitutions are hypothesized to reduce HA binding, allowing efficient release of virus with reduced NA activity. Previous studies have also demonstrated that when HA affinity is altered by glycosylation, there is a requirement for a complementary amount of activity from NA for efficient replication in tissue culture (10).

We have developed a method for measuring HA/NA balance by using a biophysical technique, bio-layer interferometry (BLI), to measure the binding and release of virus from immobilized receptor analogs on a biosensor surface in real time, allowing measurements of the kinetics of these interactions. This technique was implemented to measure the binding of viruses to receptor analogs, allowing the dissection of the role of HA and NA in controlling receptor binding. The HA/NA balance measurements were compared, measuring binding to α2,6- and α2,3-linked sialic acid receptor analogs and examining the changes in binding patterns seen when altering previously studied virus characteristics.

## EXPERIMENTAL PROCEDURES

### 

#### 

##### Viruses

Virus stocks of X-31 (a high-growth reassortant containing the HA and NA genes of strain A/Aichi/2/68 and the remaining genes of strain A/Puerto Rico/8/34) and X-31 HAM (horse adsorption mutant, X-31 with an L226Q substitution in HA) were obtained from the World Health Organization Collaborating Centre for Reference and Research on Influenza, MRC National Institute for Medical Research (London, United Kingdom). Viruses were propagated in the allantoic cavities of 11–12-day-old hen eggs. Allantoic fluid was harvested after 48–72 h. X-31 NAΔ10 was generated by cloning X-31 HA and NA from generated cDNAs into the pHW2000 vector based on a previously described system (11). Residues 60–69 of NA were deleted using a Q5 site-directed mutagenesis kit (New England Biolabs) according to the manufacturer's instructions. Recombinant viruses were generated by transfecting 293T cells with plasmids containing X-31 HA and NA and the remaining six gene segments from A/Puerto Rico/8/34. Eggs were directly infected with the transfection supernatant.

##### Virus Purification

Allantoic fluid was clarified by low-speed centrifugation. The virus was pelleted and then purified through a continuous 15–40% (w/v) sucrose gradient. Virus-containing bands were pelleted and stored in PBS and 0.01% (w/v) sodium azide at 4 °C. Virus concentration was determined by solid-phase nucleoprotein ELISA (12).

##### Virus Binding Assays

Virus binding was measured using an Octet RED system (Pall ForteBio Corp., Menlo Park, CA). The receptor analogs used were sialoglycopolymers consisting of a polyacrylamide backbone conjugated to 20 mol % sugar (α2,6-sialyl-*N*-acetyllactosamine (6SLN) or α2,3-sialyl-*N*-acetyllactosamine (3SLN)) and 5 mol % biotin (Lectinity Holdings, Moscow, Russia). Sugars were loaded onto streptavidin biosensors (Pall ForteBio Corp.) at 0.5 μg/ml for 600 s to saturate the sensors to a maximum response of ∼0.6 nm. Binding of 100 pm virus was measured for 1 h. All experiments were carried out in 10 mm HEPES (pH 7.4), 150 mm NaCl, 0.005% (v/v) Tween 20, and 4 mm CaCl_2_ at 25 °C. In some experiments, all solutions also contained both of the NA inhibitors at 100 μm (oseltamivir carboxylate (Roche Products Ltd., Welwyn Garden City, United Kingdom) and zanamivir (GlaxoSmithKline, Stevenage, United Kingdom)) to prevent cleavage of the receptor analogs by viral NA.

**FIGURE 1.**
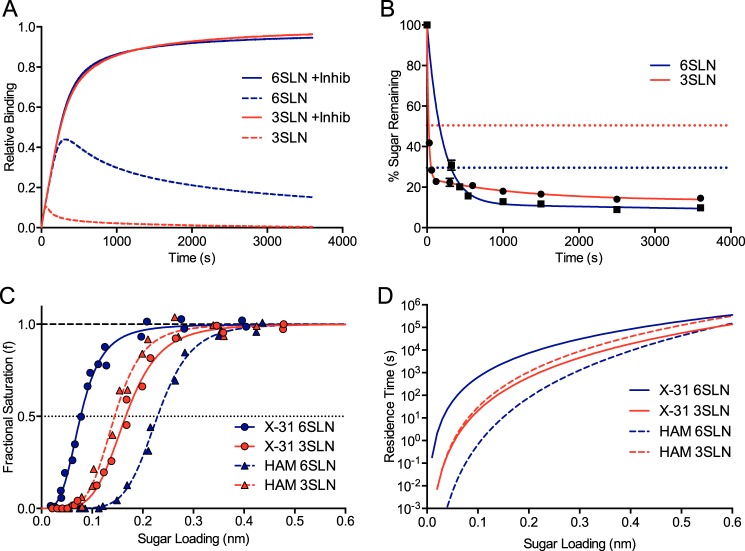
*A*, BLI curves of X-31 (100 pm) binding to sensors saturated with the receptor analogs 6SLN and linked 3SLN in the presence (*solid lines*) and absence (*dashed lines*) of NA inhibitors (*Inhib*). *B*, BLI data measuring the depletion of sugar on biosensor surfaces for X-31 binding to 6SLN and 3SLN. *Dotted lines* indicate the sugar loading value at which the fractional saturation of the sensor is 95% (*f* = 0.95), consequently the value at which sugar depletion is likely to affect HA receptor binding. Each data point is the mean of three independent measurements. *Error bars* indicate S.D. *C*, virus binding measured as a function of sugar loading in the presence of NA inhibitors for both X-31 and X-31 HAM binding to 6SLN and 3SLN. *D*, mean virus residence time determined from the data shown in *C* for X-31 and X-31 HAM binding to 6SLN and 3SLN at different sugar loadings. Mean residence time was estimated by determining 1/*k*_off_ (*k*_off_ = *k*_on_ × *K_d_*).

Virus binding data obtained in the presence of NA inhibitors underlie the calculations used here and are shown in Fig. 1*C*. These data show fractional saturation of the sensor (*f*) as a function of the relative sugar loading (RSL) and were fitted to a modified version of the Hill equation (Equation 1),

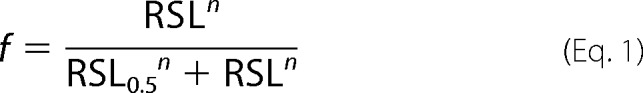
 where RSL_0.5_ is the RSL when *f* = 0.5, and *n* is the Hill coefficient. The fractional saturation values at different sugar loadings can be converted to an apparent equilibrium dissociation constant (*K_d_*) for virus binding using Equation 2.

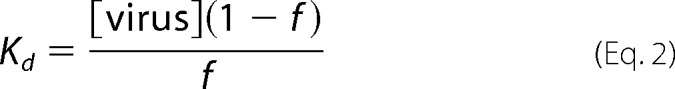


The association rate constant for virus binding (*k*_on_) in the presence of NA inhibitors was determined by measuring the observed rate for binding (*k*_obs_) as a function of virus concentration. The *k*_on_ value for X-31 and X-31 HAM was found to be ∼4 × 10^7^ m^−1^ s^−1^ for both 6SLN and 3SLN and, to a first approximation, was found to be independent of RSL. An approximate dissociation rate constant for the virus at any particular sugar loading can then be calculated (*k*_off_ = *k*_on_ × *K_d_*), and this gives the mean residence time of the virus on the sensor calculated as 1/*k*_off_.

Virus binding measurements performed in the absence of NA inhibitors were designed to assess how rapidly NA could cleave sugars on the sensor surface. These sugar depletion experiments were carried out by allowing the virus to bind in the absence of NA inhibitors for different preset times (ranging from 30 to 3600 s), after which NA inhibitors (100 μm) were added, and the association was allowed to proceed to completion. Dividing the amplitude of the final response by the maximum response observed in experiments performed in the presence of NA inhibitors gave the fractional saturation value (*f*). These *f* values for the different preset times were then converted to RSL values using Equation 1 rearranged as follows (Equation 3).

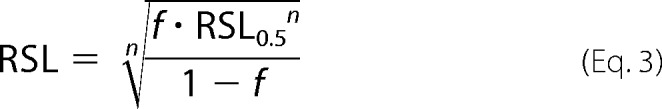
 These RSL values were then converted to a percentage of initial saturated loading (0.6 nm) and plotted as a function of time.

##### NA Kinetics

NA was purified from bromelain-treated purified virus as described previously (13). Enzymatic parameters for the NA substrate 2′-(4-methylumbelliferyl)-α-d-*N*-acetylneuraminic acid (MUNANA; Sigma) were measured in a continuous assay in a Jasco FP-6300 spectrofluorometer as described previously (14). MUNANA concentrations ranging from 5 to 75 μm were used. Measurements were made in 10 mm HEPES (pH 7.4), 150 mm NaCl, 0.005% (v/v) Tween 20, and 4 mm CaCl_2_ at 37 °C. NA kinetic parameters for the natural substrates 6SLN and 3SLN (Dextra Laboratories) were determined using an assay previously used to measure IAV NA kinetics (15). This assay uses β-galactosidase (Sigma) and β-galactose dehydrogenase (Roche Products Ltd.) as reporter enzymes. Each 100-μl reaction contained 25 units of β-galactosidase, 0.25 units of β-galactose dehydrogenase, and 5 mm NAD^+^ (Sigma) in 10 mm HEPES (pH 7.4), 150 mm NaCl, 0.005% (v/v) Tween 20, 4 mm CaCl_2_, and 1 mm MgCl_2_ at 37 °C. Reactions were monitored by measuring absorbance at 340 nm in a 10-mm path length cuvette using a Jasco V-550 spectrometer, and initial reaction rates were measured. This change in absorbance was converted to change in concentration using the extinction coefficient of NADH (ϵ = 6220 m^−1^ cm^−1^). The results of kinetic assays were fitted to the Michaelis-Menten equation using GraphPad Prism 6 (GraphPad Software, San Diego, CA).

## RESULTS

### 

#### 

##### Measurement of Influenza HA/NA Balance

To study HA/NA balance, we used a modified version of a previously published virus binding assay using BLI (12). This modified assay measures virus binding to receptor analogs in the presence and absence of NA inhibitors. Fig. 1*A* shows the results from this assay measuring the interaction of a 1968 pandemic high-growth reassortant H3N2 virus (X-31) on sensors saturated with human α2,6-linked and avian α2,3-linked sialic acid receptor analogs (6SLN and 3SLN). The two curves with inhibited NA are very similar in shape. Initially, the virus binding with uninhibited NA parallels the virus binding with inhibited NA, but after a short time, the curves for the virus with uninhibited NA reverse direction, exhibiting an inflection point, and eventually decay to very low virus binding levels. Curves for X-31 binding to 6SLN and 3SLN in the absence of NA inhibitors are similar in shape, but the overall binding level is much lower with 3SLN. Similar curve shapes are found when inhibiting viral NA and complementing the solution with soluble bacterial NA (data not shown). The striking observation that the curves for the virus with uninhibited NA reverse direction at the point where the control curves (virus with inhibited NA) indicate that the sensor surface is only partially saturated with virus suggests that uninhibited NA is able to rapidly remove substantial amounts of sugar from the sensor surface.

The difference between the two curves (inhibited and uninhibited) must of course be due to the action of NA. To determine the effect of NA on surface-immobilized sugars, we estimated the extent to which sugar became depleted as a function of time by measuring the remaining capacity of the sensor to bind the virus at various set time points (see “Experimental Procedures”). These data for X-31 are shown in Fig. 1*B*. For both receptor analogs, there was an initial rapid depletion of sialic acid, followed by a much slower depletion. Fig. 1*B* also shows the sugar loading value that corresponds to a fractional saturation value of 0.95 (determined from data shown in Fig. 1*C*). This sugar loading value was then converted to the percentage of sialic acid remaining using an initial sugar loading response of 0.6 nm. This value is the depletion value at which the sialic acid will have been removed to such an extent that the virus is no longer able to completely saturate the surface. For both receptor analogs, the point at which the depletion curve crosses this line corresponds to the time where there is significant deviation between the inhibited and uninhibited binding curves. The initial rapid depletion took place surprisingly quickly for both receptors, with >50% of 3SLN cleaved by 30 s and >50% of 6SLN by 320 s. The initial sugar depletion was much faster for 3SLN; however, the amount of sugar remaining on the surface after long times was higher compared with 6SLN. This is likely due to the lower binding of X-31 to 3SLN at lower sugar loadings, thus preventing NA from acting on any remaining receptor (Fig. 1*C*).

The virus binding signals measure directly how the virus interacts with and alters the sensor surface. The affinity of X-31 HA for monomeric sugars is extremely low (16): *K_d_* = 2.1 ± 0.3 mm for 6SLN and 3.2 ± 0.6 mm for 3SLN (17). The interaction of a virus with a sugar-containing surface is multivalent, and the avidity effect means that virus affinity is high, and consequently, the *K_d_* is very low. Virus binding affinities can be estimated by measuring the fractional saturation of a sensor surface at a fixed concentration of virus as a function of the sugar loading (Fig. 1*C*). These fractional saturation data can then be converted to apparent *K_d_* values. For example, we estimated the *K_d_* values of X-31 for 6SLN (3SLN) to be 1 nm at a sugar loading of 0.038 (0.076) nm, 100 pm at 0.08 (0.165) nm, and 10 pm at 0.155 (0.28) nm. These apparent *K_d_* values can be converted to estimate the mean virus residence time on the sensor by calculating 1/*k*_off_ at different sugar loadings, shown in Fig. 1*D* (see “Experimental Procedures”).

Kinetic parameters for X-31 NA were measured to determine the ability of NA to cleave different substrates: the fluorogenic substrate MUNANA and the natural substrates 6SLN and 3SLN (Table 1). X-31 NA showed a much higher efficiency for cleaving 3SLN, with an ∼15-fold lower *K_m_* and ∼5-fold higher turnover rate (*k*_cat_). This preference of NA for α2,3-linked sialic acid is likely due to the inherent property of the ketosidic bond between the sialic acid and the galactose, which is inherently more cleavable in the α2,3-linked form.

**TABLE 1:** **Steady-state kinetic parameters determined for purified X-31 NA**

Substrate	*K_m_* (mean ± S.E.)	*k*_cat_(mean ± S.E.)	*k*_cat_/*K_m_*
	μ*m*	*s*^−*1*^	μ*m*^−*1*^ *s*^−*1*^
MUNANA	27.6 ± 1.0	56.6 ± 0.9	2.05
6SLN	8070 ± 615	18.5 ± 0.9	0.00229
3SLN	562.3 ± 20	97.5 ± 1.5	0.173

##### Effect of Altering HA Avidity

To investigate the effect of altering HA properties, measurements were made on the previously characterized X-31 mutant X-31 HAM. This virus, selected by passage of X-31 in the presence of horse serum (18), contains the L226Q substitution in HA.

**FIGURE 2.**
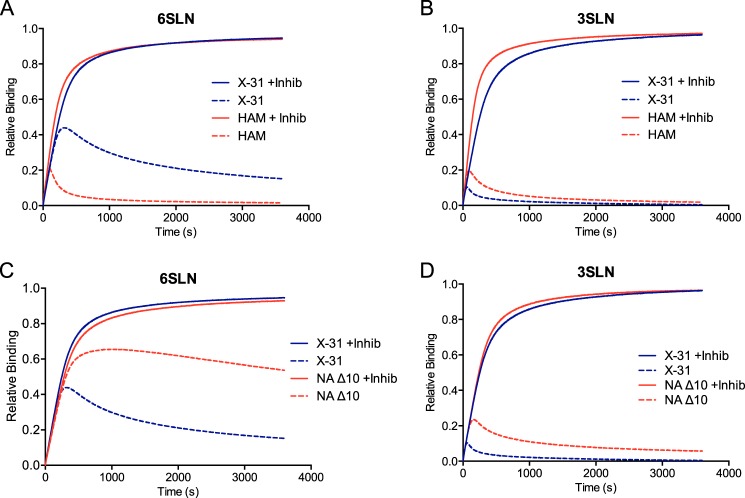
**BLI data for binding of X-31 (100 pm) and associated mutant viruses to sensors saturated with receptor analogs 6SLN and 3SLN in the presence (*solid lines*) and absence (*dashed lines*) of NA inhibitors (*Inhib*).** Shown are X-31 and X-31 HAM binding to 6SLN (*A*) and 3SLN (*B*) and X-31 and X-31 NAΔ10 binding to 6SLN (*C*) and 3SLN (*D*).

This mutation reduces the affinity of HA for monomeric 6SLN (*K_d_* = 5.9 ± 0.7 mm) but has little effect on the affinity of HA for 3SLN (*K_d_* = 2.9 ± 0.3 mm) (17). These changes are reflected, as expected, in the affinity of the virus for sugar-coated surfaces. Virus affinity for 6SLN-coated surfaces was significantly lower compared with X-31, whereas the affinity for 3SLN-coated surfaces was slightly higher (Fig. 1*C*). Compared with X-31, this virus has an apparent *K_d_* of 100 pm at a sugar loading of 0.144 nm for 3SLN (0.165 nm for X-31) and 0.23 nm for 6SLN (0.08 nm for X-31). The HA/NA balance of X-31 HAM was compared with that of X-31 (Fig. 2, *A* and *B*). The changes in receptor binding affinity resulted in significant changes in the shape of the binding curve measured with uninhibited NA. The overall binding to 6SLN was reduced dramatically, with the inhibited and uninhibited curves diverging much earlier. The small increase in 3SLN receptor binding appears to result in a slight increase in overall virus binding with uninhibited NA.

##### Effect of Altering NA Properties

NA stalk length has previously been shown to change upon adaption of viruses to new hosts, altering virus growth characteristics (19). An X-31 virus with residues 60–69 deleted in the NA stalk (X-31 NAΔ10) was generated by reverse genetics. Receptor binding was measured and compared with that observed with X-31 (Fig. 2, *C* and *D*). The NA activity cleaving the small substrate MUNANA remained unchanged for both the wild-type and shortened stalks (data not shown), indicating that the sialidase activity of NA is unchanged. The deletion of 10 residues from the NA stalk caused major changes in the binding curves (with uninhibited NA) for both 6SLN and 3SLN. The point of inflection occurred at later times, and the overall binding levels were increased. This is presumably due to a reduction in the ability of NA to cleave the immobilized substrate, as the reduction in stalk length will move the catalytic region of NA closer to the viral membrane, reducing the ability of NA to form an enzyme-substrate complex, without any changes in the catalytic properties of the enzyme.

## DISCUSSION

It is clear from the different curves shown in Figs. 1 (*A* and *B*) and 2 that the observed behavior of virus binding and release from a surface depends critically on the balance between the affinity of viral HA for the receptor and the ability of viral NA to cleave sugars from the surface. Three features of the curves for virus binding with uninhibited NA need to be explained: the rapid curve inflection, the slow subsequent release of bound virus, and the fact that some virus remains bound to the surface even after very long times. The data for binding of X-31 to 3SLN with uninhibited NA show a very fast curve inflection, reaching a maximum saturation of only ∼10% of that seen in experiments with inhibited NA. This indicates that viruses must be making short interactions with the surface during which large amounts of sugar are cleaved. Because the interaction of HA with individual sialic acids is very weak (*K_d_* in the mm range), the virus must form several HA-sialic acid interactions to create a stable long-lived interaction. There is consequently likely to be a number of short-lived transient interactions of the virus with the sensor before a strong interaction is eventually formed. When an individual virus makes contact with the sensor surface, there will be a number of factors that will determine the strength (and therefore, the duration) of the interaction, including the number and distribution of HAs and sugar ligands in the interaction area. Without knowing the exact nature of these transient interactions, it is difficult to know what the mean residence time might be. One can speculate that the interaction could be similar in strength to that seen (with inhibited NA) at low sugar loadings. For example, we estimated that a sugar loading of 0.02 nm would correspond to residence times of ∼30–50 ms for 3SLN and ∼2–5 s for 6SLN (Fig. 1*D*). The question then is how much sugar NA can cleave in these times. This will depend on the positions and distribution of NAs on the virion, the positioning and distribution of the substrate, and, most importantly, on the catalytic efficiency (*k*_cat_/*K_m_*) of the enzyme for the two substrates. These values indicate that NA cleaves α2,3-linked sugars much more efficiently than α2,6-linked sugars. This large difference in catalytic efficiency explains how X-31 can deplete 3SLN much faster even though the residence time of the virus is less than for 6SLN. In comparing X-31 HAM with X-31, the much faster inflection point (and reduced overall binding) observed for X-31 HAM interacting with the 6SLN-coated surface is attributable to the reduced virus avidity and correspondingly shorter residence time. In comparing the stalk deletion mutant with X-31, the much longer turnaround time (and increased overall binding) observed for the mutant interacting with both the 6SLN- and 3SLN-coated surfaces is attributable to the reduced ability of NA to cleave sugars despite a fully active sialidase.

The second binding phase (following the point where the binding curves turn downwards) presumably reflects the rate of dissociation of those viruses that had become strongly attached during the initial binding phase. Because strongly attached viruses would normally have very long residence times (Fig. 1*D*), the different behavior observed for the two receptor analogs must be due solely to the properties of NA. The observation that the second binding phase observed for X-31 is faster with the 3SLN-coated surface (Fig. 1*A*) is consistent with the greater catalytic efficiency of the enzyme for this substrate. The fact that the second phase is much slower for the stalk mutant with both sugars is also consistent with the idea that this phase depends strongly on the properties of NA (Fig. 2, *C* and *D*).

The slow phase observed for X-31 binding to 6SLN with uninhibited NA (Fig. 1*A*) does not reach zero (no virus bound) even at very long times, indicating that some virus is still interacting with the sensor surface. There are two possible explanations for this: 1) a population of essentially irreversibly attached virus or 2) the viruses interacting with the surface are attaching in a very transient manner that does not allow NA to efficiently meet substrate. The measurements of sugar depletion for both 6SLN and 3SLN tend toward a value of ∼10% remaining sugar (Fig. 1*B*), which equates to a sugar loading of ∼0.06 nm. At this value, the difference in mean virus residence times is ∼100 s for 6SLN and ∼1.7 s for 3SLN. This residence time appears not to allow for the hypothesis of irreversibly attached virus, rather that the interaction with the surface is more transient. There is also limited ability for NA to cleave 6SLN, with its lower catalytic efficiency.

An interesting finding from the data presented is the large difference in uninhibited X-31 binding to α2,6- and α2,3-linked sialic acids. These data show that the substrate specificity of NA can have a large impact on virus receptor binding. A number of reports in the past indicate that IAV NAs have a preference for cleaving α2,3-linked sialic acid (3, 20–22). Whether this is an evolved enzymatic property or an inherent property of the chemical bond is unclear. The implications for this linkage preference are interesting when comparing avian and human viruses. The primary site for human virus infection is in the upper respiratory tract, with the virus infecting cells mainly expressing α2,6-linked sialic acid (23). Mucus, which is reported to be rich in α2,3-linked sialic acid, is present in the upper respiratory tract (24). For a human virus to replicate efficiently, it needs to have a combination of HA and NA activities that allow it to efficiently escape nonspecific inhibition by mucins to efficiently infect the target cells. To avoid the inhibition of the mucus, the virus can either have HA with low avidity for α2,3-linked sialic acid, NA with greater activity against α2,3-linked sialic acid, or a combination of the two. For efficient cell attachment and viral entry, there is a requirement for a human virus to have a strong avidity for α2,6-linked sugars, and for virus release and prevention of aggregation following virus egress from the cell, there will be a requirement for efficient cleavage of 2,6-linked sugars.

The system for measuring HA/NA balance described here involves measuring the solid-phase binding of a relatively high virus concentration (100 pm ≈ 6 × 10^10^ particles/ml). This quantity of virus is much higher than that in natural infections, which are thought to be initiated by a relatively small number of virus particles. Nevertheless, these experiments can be used to infer characteristics of cell attachment and virus release. This method is useful to assess new and emerging viral strains and to understand their relative HA/NA balance characteristics and compare these with previously measured pandemic strains. This provides much more information when studying virus characteristics over and above simply measuring HA or NA kinetic parameters in isolation, allowing opportunities to study the complex relationship between HA and NA, which involves many aspects of cooperativity and multivalency.

## Footnotes

2 The abbreviations used are:

IAVs: influenza A viruses
HA: hemagglutinin
NA: neuraminidase
BLI: bio-layer interferometry
6SLN: α2,6-sialyl-*N*-acetyllactosamine
3SLN: α2,3-sialyl-*N*-acetyllactosamine
RSL: relative sugar loading
MUNANA: 2′-(4-methylumbelliferyl)-α-d-*N*-acetylneuraminic acid.
